# pH Transitions and electrochemical behavior during the synthesis of iron oxide nanoparticles with gas-diffusion electrodes†

**DOI:** 10.1039/c9na00738e

**Published:** 2020-02-13

**Authors:** Rutely C. Burgos-Castillo, Arturo Garcia-Mendoza, Yolanda Alvarez-Gallego, Jan Fransaer, Mika Sillanpää, Xochitl Dominguez-Benetton

**Affiliations:** Separation and Conversion Technologies, Flemish Institute for Technological Research (VITO) Boeretang 200 2400 Mol Belgium xoch@vito.be rcbcastillo@gmail.com; Department of Green Chemistry, School of Engineering Science, Lappeenranta University of Technology Sammonkatu 12 FI-50130 Mikkeli Finland; Departamento de Química Analítica, Facultad de Química, Universidad Nacional Autónoma de México Av. Universidad 3000, C.U Mexico City 04510 Mexico; SIM vzw Technologiepark 935 BE-9052 Zwijnaarde Belgium; Department of Materials Engineering, Katholieke Universiteit Leuven (KU Leuven) Kasteelpark Arenberg 44 - bus 2450 B-3001 Leuven Belgium

## Abstract

Gas diffusion electrocrystallization (GDEx) was explored for the synthesis of iron oxide nanoparticles (IONPs). A gas-diffusion cathode was employed to reduce oxygen, producing hydroxyl ions (OH^−^) and oxidants (H_2_O_2_ and HO_2_^−^), which acted as reactive intermediates for the formation of stable IONPs. The IONPs were mainly composed of pure magnetite. However, their composition strongly depended on the presence of a weak acid, *i.e.*, ammonium chloride (NH_4_Cl), and on the applied electrode potential. Pure magnetite was obtained due to the simultaneous action of H_2_O_2_ and the buffer capacity of the added NH_4_Cl. Magnetite and goethite were identified as products under different operating conditions. The presence of NH_4_Cl facilitated an acid–base reaction and, in some cases, led to cathodic deprotonation, forming a surplus of hydrogen peroxide, while adding the weak acid promoted gradual changes in the pH by slightly enhancing H_2_O_2_ production when increasing the applied potential. This also resulted in smaller average crystallite sizes as follows: 20.3 ± 0.6 at −0.350 V, 14.7 ± 2.1 at −0.550 and 12.0 ± 2.0 at −0.750 V. GDEx is also demonstrated to be a green, effective, and efficient cathodic process to recover soluble iron to IONPs, being capable of removing >99% of the iron initially present in the solution.

## Abstract 2

Gas diffusion electrocrystallization (GDEx) was explored for the synthesis of iron oxide nanoparticles (IONPs).

## Graphical abstract

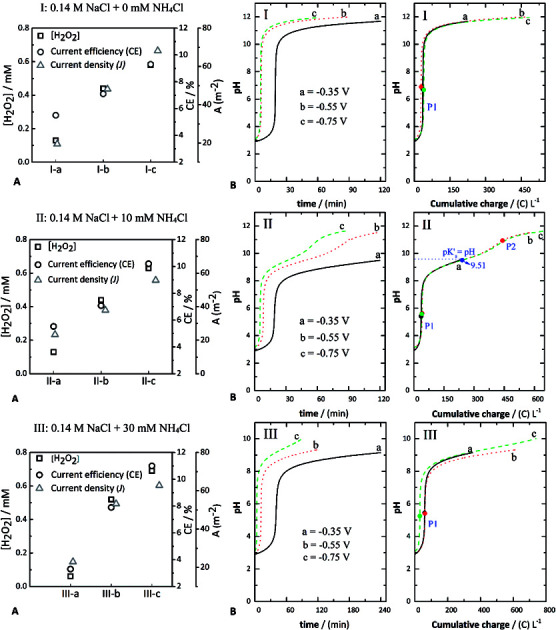

## Introduction

Nanoparticle production methods that are economical, clean, safe, easy-to-implement, and upscalable are an active research subject.^1–3^ A key disadvantage of most alternatives^4–7^ is the use of non-aqueous molecular solvents and reducing and capping agents, which are often environmentally hazardous and added in excess.^8,9^ Moreover, most methods involve several processing steps, often including different upstream and downstream units, which can appear to be suitable at the lab scale but are challenging to implement industrially.^5,10–12^

The development of environmentally friendly synthesis methods has gained particular attention,^13–17^ intensifying the interest in electrochemistry as a synthetic platform under mild conditions.^18–25^ In most instances, known electrochemical approaches for the synthesis of nanoparticles^26–28^ involve the growth of films onto inert substrates, mostly by one-step electrodeposition, at working temperatures between 70° and 90 °C. A compilation of pathways and methods for the electrochemical formation of magnetite nanoparticles is available in the scientific literature.^23,26,29–32^

In the present study, a new electrochemical method, called gas-diffusion electrocrystallization (GDEx),^14^ is employed for the formation of iron oxide nanoparticles (IONPs)^33^ from a soluble iron precursor (Fe^2+^). A gas diffusion cathode is used for the electroreduction of oxygen (O_2_) contained in a gas phase (air) which, in turn, drives the precipitation of crystalline IONPs at the electrochemical interface.

The oxygen reduction reaction (ORR) using carbon-based gas-diffusion electrodes has been thoroughly investigated and well demonstrated in acidic and alkaline media. Reactions (1) and (2) show the two-electron ORR of O_2_ to H_2_O_2_ or hydroperoxide ions (HO_2_^−^)^41^ and occur under acidic and alkaline conditions, respectively. Besides, under alkaline conditions, HO_2_^−^ can be reduced to OH^−^*via* reaction (3).

1 O_2_ + 2H^+^ + 2e^−^ → H_2_O_2_

2 O_2_ + H_2_O + 2e^−^ → HO_2_^−^ + OH^−^

3 HO_2_^−^ + H_2_O + 2e^−^ → 3OH^−^

Throughout this work, both chemical species, *i.e.*, H_2_O_2_ and HO_2_^−^, will be referred to as H_2_O_2_, granting that at more alkaline pHs HO_2_^−^ will be the most abundant species (p*K*_a_ = 11.65).^24,34^ The effective dissolution of O_2_ gas is the limiting step of this process, but with gas-diffusion electrodes this is overcome by the high mass transport rates at the triple-phase boundary.^35^

The present work differs from previous electrochemical methods in various ways. First, it employs a gas-diffusion cathode to drive the formation of IONPs. Thus, the precipitating agents (OH^−^) and H_2_O_2_ are produced *in situ via* the ORR. The synthesis takes place in an aqueous solution with a supporting electrolyte containing no other additives or surfactants, over a range of applied potentials between −0.350 V and −0.750 V *versus* a reference Ag/AgCl (saturated KCl) electrode. Furthermore, our method does not require a high temperature, and it involves only one synthesis step. An understanding of GDEx is incipient. Thus, the present research attempts to reveal relevant phenomenological and mechanistic events to establish it as a prominent green route to fabricating nanoparticles.

The first purpose of this work was to identify pH transitions imposed by the ORR products (*i.e.*, OH^−^ and H_2_O_2_) in the catholyte bulk, as well as the electrochemical behaviour (current–potential response) of the system. In addition to investigating the unbuffered system, the effect of adding a weak acid (WA) was studied, hypothesizing that the WA would serve as a co-catalyst for the ORR and, hence, the formation of H_2_O_2_. Our key aim was to study this in the presence of a soluble iron precursor, for its recovery to NPs. Furthermore, the correlation between the yield of H_2_O_2_ and the recovery rate of iron upon the addition of a WA (*i.e.*, NH_4_Cl) was studied. Ultimately, the influence of adding a WA on the production of NPs was assessed.

## Experimental

### Materials and methods

#### Reagents

Iron chloride (FeCl_2_), sodium chloride (NaCl), ammonium chloride (NH_4_Cl), and hydrogen peroxide (H_2_O_2_, 30 wt%) were of analytical grade, purchased from Merck, Sigma Aldrich, Fluka, and Acros Organics, respectively. Concentrated hydrochloric acid (HCl, 37 wt%) was of analytical grade and purchased from Panreac and Acros Organics. Other chemicals used for analysis were of analytical grade from Fluka, Sigma-Aldrich, and Panreac. All the reagents were used as received. Solutions were prepared with Millipore Milli-Q water (resistivity > 18 MΩ cm)

#### Electrochemical set-up

The electrochemical set-up consisted of two cylindrical compartments (20 mL) separated by an ion-permeable porous separator (ZIRFON® PERL, Agfa Gevaert).^36^ A dual peristaltic pump was used to recirculate the electrolytes^15^ at 40 mL min^−1^ from two independent external electrolyte reservoirs, each containing 250 mL of the electrolyte (*i.e.*, anolyte and catholyte, respectively).

The reference electrode was a Ag/AgCl electrode (satd. KCl, Biologic, model: R-XR300). All potentials measured in this work are reported *versus* its potential. The anode (counter electrode) was a platinum disc of a 10 cm^2^ projected surface area, which was laser welded onto a titanium plate. This anode generated O_2_ or Cl_2_, which had a negligible influence on the electrochemical (cathodic) process and products of interest. The cathode was a cold-rolled gas-diffusion electrode (VITO CORE®) composed of a hydrophilic active carbon-polytetrafluoroethylene layer (C-PTFE with a C to PTFE weight ratio of 80 : 20) pressed in between stainless steel mesh serving as a current collector and a hydrophobic PTFE layer through which oxygen percolated.^15^ The active carbon was Norit® SX 1G (Cabot, Europe). The projected surface area of the cathode was 10 cm^2^.^15^ Air was supplied (as the source of O_2_ gas) at 200 mL min^−1^ using a mass flow controller at an overpressure of 30 mbar (g).

#### Electrogeneration of H_2_O_2_

Reduction of O_2_ was conducted at different cathode potentials (−0.350 V, −0.550 V, and −0.750 V *vs.* Ag/AgCl) provided using a multi-channel potentiostat/galvanostat (VMP-3, Bio-Logic SAS, France). The current, the working electrode potential, the charge, the cell potential, and the pH evolution of the 250 mL recirculated solution were recorded during the assays in 0.1 M NaCl as the background electrolyte. The pH evolution was mainly controlled by the continuous electrogeneration of hydroxide ions (OH^−^), although hydrogen peroxide decomposition can also play a role.^44^ The electrogenerated H_2_O_2_ was measured using the iodide method.^37,38^ Aliquots (0.5 mL) of electrolyzed solutions (without ferrous salts) were added to a mixture of 2.5 mL of 0.1 M potassium phthalate + 2.5 mL of the iodide reagent (0.4 M potassium iodide, 0.06 M NaOH, and ∼10^−4^ M ammonium molybdate) and diluted to 10 mL to be within the linearity of the calibration curve. The samples were analysed using a UV-vis spectrometer after 15 min of reaction. In a few experiments, the concentration of H_2_O_2_ was monitored using test strip analysis (QUANTOFIX® Relax). The current efficiency (CE(%)), that is the ratio between the charge used for producing H_2_O_2_ and the total electrochemical charge passed during the electrolysis, was determined using eqn (4):4
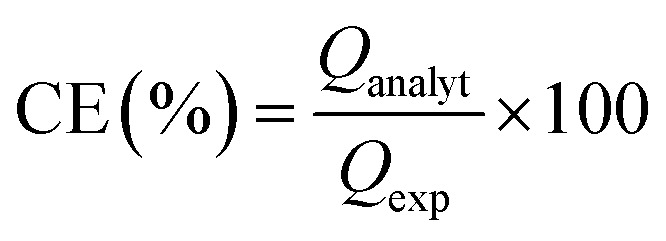
where *Q*_analyt_ (C) is the effective charge that produces H_2_O_2_ (measured analytically). *Q*_exp_ is the total charge (C) that is passed through the electrochemical circuit during each electrolysis, as explained in Table S3, ESI†.

#### Synthesis of IONPs

IONPs were synthesized at room temperature (∼18 ± 0.2 °C) as previously described.^33^ Hydroxyl ions and hydrogen peroxide produced in the gas-diffusion cathode encounter Fe^2+^ ions at the surface of the gas-diffusion electrode, resulting in the formation of IONPs. Solutions of a 250 mL background electrolyte, *i.e.*, 0.140 M NaCl, supplemented with FeCl_2_, with and without NH_4_Cl, were processed from an initial pH ∼2.7 to a pH above 7. After the conclusion of each experiment, the suspensions were allowed to settle in the same reaction medium under stagnation conditions. Afterward, the supernatant of each suspension was decanted. Next, the resulting sediment was centrifuged at 3200 rpm for 15 min at room temperature to eliminate the remaining water, forming a pellet. The latter was cleaned at least twice with distilled water or more if needed and then dried at room temperature under a nitrogen gas atmosphere.

#### X-ray diffraction

The diffraction patterns of all the samples were obtained *via* X-ray diffraction (XRD, PANalytical Empyrean) with Co Kα radiation (*λ* = 1.78091 Å), operated at 40 kV. The average crystallite size was calculated using the Scherrer equation (5),^39^ as follows:5
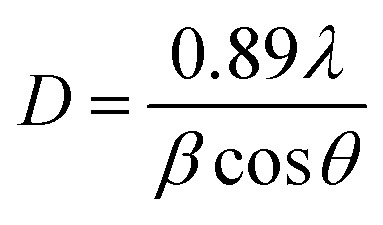
where 0.89 is the shape factor for spherical particles, *D* is the average crystallite size, *λ* is the X-ray wavelength (*λ* = 1.78091 Å), *β* is the line broadening at half the maximum intensity (FWHM) in radians, and *θ* (°) is the Bragg angle for a reflecting plane. All measurements were performed at room temperature with a fixed divergence slit of 0.76 nm in the 2*θ* range of 5–120°, with a step size of 0.01313° and time per scan of 48.195 s.

#### Current–potential curves

Current–potential curves were recorded by linear sweep voltammetry (LSV) to examine the electrocatalytic activity for the electroreduction of O_2_ with and without the addition of iron, at a scan rate of 10 mV s^−1^ using a multi-channel potentiostat/galvanostat (VMP-3, Bio-Logic SAS, France).

## Results and discussion

### Control electrolytes without Fe^2+^

The aim of this part of the study was two-fold: (i) to establish the capability of the process to accumulate H_2_O_2_ in the absence of metal ions (*i.e.*, Fe^2+^) and (ii) to identify the consumed charge to transform the pH from acidic (2.7–3) to alkaline conditions (pH 8–12). Three different electrolyte compositions were studied at applied potentials of −0.350 V (a); −0.550 V (b); and −0.750 V (c). The conditions of the control experiments are outlined in Table 1: 0.14 M NaCl (assays I-a, I-b, and I-c), 0.14 M NaCl + 10 mM NH_4_Cl (assays II-a, II-b, and II-c), 0.14 M NaCl + 30 mM NH_4_Cl (assays III-a, III-b, and III-c). For comparison purposes between experiments, a one-hour period was chosen to measure the concentration of H_2_O_2_, which was then normalized to the amount of charge consumed for each experiment from which the corresponding electrolyte samples were extracted. In all instances, this allowed sufficient evolution to reach the target pH.

**Table 1:** Reaction conditions during the pH evolution by the control electrolytes without Fe^2+^

Assay	Electrolyte	Applied potential (V)
I-a	140 mM NaCl	−0.350
I-b	140 mM NaCl	−0.550
I-c	140 mM NaCl	−0.750
II-a	140 mM NaCl + 10 mM NH_4_Cl	−0.350
II-b	140 mM NaCl + 10 mM NH_4_Cl	−0.550
II-c	140 mM NaCl + 10 mM NH_4_Cl	−0.750
III-a	140 mM NaCl + 30 mM NH_4_Cl	−0.350
III-b	140 mM NaCl + 30 mM NH_4_Cl	−0.550
III-c	140 mM NaCl + 30 mM NH_4_Cl	−0.750

### pH evolution

To assess the evolution of pH and accumulation of H_2_O_2_—during the electroreduction of O_2_—the applied potential was varied for each experiment, as stated above. In addition, two different concentrations of NH_4_Cl (p*K*_a_ = 9.26) were tested and compared to the case without the WA. Given the theory of cathodic deprotonation of WAs,^37,39,40^ it was expected that the addition of NH_4_Cl would induce a co-catalytic effect on the electroreduction of O_2_ to H_2_O_2_.^41–43^

**Fig. 1:**
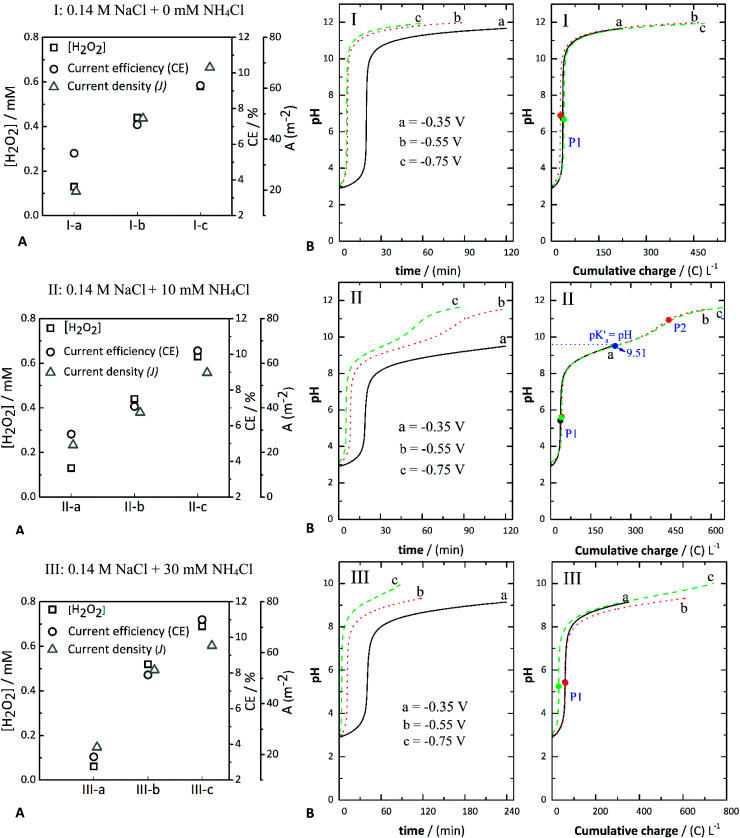
Concentration of H_2_O_2_, current efficiency (CE%) and current density (*J*) (A) and evolution of pH (B) against either time or charge consumed in: 0.14 M NaCl (I-a, I-b, and I-c), 0.14 M NaCl + 10 mM NH_4_Cl (II-a, II-b, and II-c), and 0.14 M NaCl + 30 mM NH_4_Cl (III-a, III-b, and III-c) at different applied potentials of −0.350 V (a), −0.550 V (b), and −0.750 V (c).

Fig. 1 depicts the evolution of pH against either time or the charge consumed for the control experiments using NaCl as the background electrolyte, *i.e.,* without addition of the metal precursor. The pH increase is attributed to the generated OH^−^ ions in the active cathode layer during the electrolytic process.^29^ Such ions diffuse towards the bulk electrolyte, increasing both the local pH at the electrochemical interface and the pH of the bulk liquid. The plots depicted for systems I-a, I-b, and I-c are comparable to the chemical titration of a strong acid, *i.e.*, HCl with a strong base (OH^−^). The OH^−^ ions formed in the GDE first react with the H_3_O^+^ ions generated from the dissociation of the HCl added to adjust the initial pH (∼3) of the solution. Notably, the pH evolved faster as the applied potential increased, *i.e.*, at either −0.55 V or −0.750 V (I-b and I-c), and as the current density increased correspondingly.

**Fig. 2:**
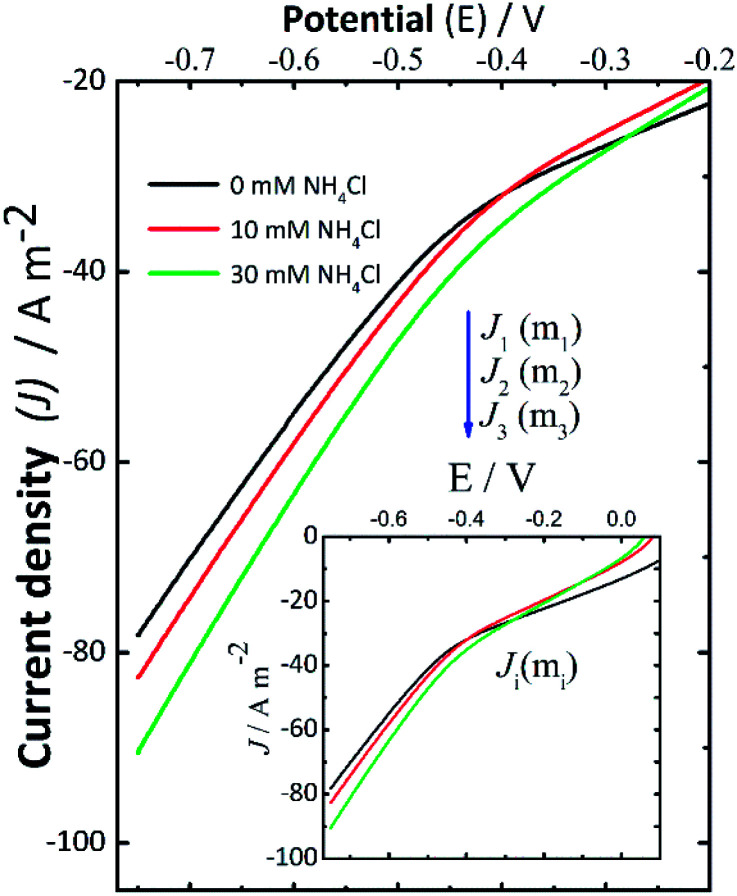
Current–potential curves recorded before starting the electrolysis by linear scan voltammetry (LSV) at a scan rate of 10 mV s^−1^ varying the polarization potential from open circuit potential to −0.75 V *vs.* Ag/AgCl (satd. KCl) in different background electrolytes: 0.14 M NaCl (black solid line), 0.14 M NaCl + 10 mM NH_4_Cl (red solid line), and 0.14 M NaCl + 30 mM NH_4_Cl (green solid line). The inset shows the full current–potential curves.

The presence of NH_4_Cl influenced the evolution of pH. Its effect can be observed by comparing the systems without (series of assays “I,” Fig. 1(I)) and with NH_4_Cl (Fig. 2(II) and (III)). When NH_4_Cl is present, two buffer regions can be differentiated, corresponding to stepwise neutralization of the different protonated species present in the electrolyte (*i.e.*, HCl and NH_4_Cl). The equivalence point of each region is identified as *P*_1_ and *P*_2_, respectively, in the plot of pH against charge (Fig. 1). In Fig. 1, the equivalence points are marked with black, red, or green circles. Another characteristic point is the pH value halfway (blue marker circles denoted as p*K*′_a_) between the charge consumed to go from *P*_1_ to *P*_2_, in which the OH^−^ equivalents that have reacted equal one-half of the WA equivalents initially present in the solution. In other words, at this point the concentration of the WA (NH_4_^+^) and its conjugate base (NH_3_) should be equal; thus, the corresponding pH should be the p*K*_a_ of the neutralized acid, in this case, NH_4_^+^.

Therefore, the value of pH at this point should coincide with the theoretical p*K*_a_ of NH_4_^+^ (∼9.2). However, the experimental value lies around p*K*′_a_ = 9.51 (Fig. 1(II)). These p*K*′_a_ values vary mainly due to reactions that occur at the same time as the equilibrium of NH_4_^+^, *e.g.*, reactions (7) and (8). The pH *versus* cumulative charge curves for the case when 30 mM NH_4_Cl is added are shown in Fig. 1(III). As more NH_4_Cl is present, the buffer region becomes larger. In contrast, the plateau length decreases when increasing the potential (from −0.35 V and −0.75 V) at a given concentration of NH_4_Cl. It should be noted that the pH values at *P*_1_ seem to be influenced by the applied potential, decreasing at more negative applied potentials. The more negative potentials increased the production of OH^−^ ions. The pH *versus* cumulative charge curves for the case when 30 mM NH_4_Cl is added are shown in Fig. 1(III). As more NH_4_Cl is present, the buffer region becomes larger. In contrast, the plateau length decreases when increasing the potential (from −0.35 V and −0.75 V) at a given concentration of NH_4_Cl. It should be noted that the pH values at *P*_1_ seem to be influenced by the applied potential, decreasing at more negative applied potentials.

The experimental pH curves *versus* time were fitted using a polynomial model to elucidate the reactions occurring in the cathodic compartment and that are likely to explain the titration curves presented in Fig. 1. These fitted curves are depicted in Fig. S4 (ESI†). As a result, it was confirmed that reaction (6) is involved in the reduction of oxygen at the cathode.

6 O_2_ + 2H_2_O + 2e^−^ ⇌ 2HO^−^ + H_2_O_2_

7 H_3_O^+^ + OH^−^ ⇌ 2H_2_O

8 NH_4_^+^ + OH^−^ ⇌ NH_3_ + H_2_O

From the experimental data, the following phenomena can be highlighted: (1) Reactions (7) and (8) occurred in sequence during the electrolytic ORR when NH_4_Cl was added. In its absence, only the ORR of reaction (6) took place, resulting in an OH^−^ titration. Therefore in the assays containing only NaCl as the background electrolyte (Fig. 1(I)), only one equivalence point was expected (circle markers in Fig. 1(I)), denoted as *P*_1_. The pH value was thus established by the action of water acting as an amphoteric molecular solvent, meaning that at *P*_1_ the number of moles of H_3_O^+^ and of OH^−^ ions is equal. (2) The pH value expected in the absence of NH_4_Cl was around seven, in close agreement with the values calculated from the experimental pH data described in Fig. 1, being 6.72 at −0.350 V (assay I-a), 6.89 at −0.550 V (assay I-b), and 6.67 at −0.750 V (assay I-c). (3) In the assays, including NaCl + NH_4_Cl, as previously discussed, two equivalence points were identified and marked as *P*_1_ and *P*_2_ (Fig. 1(II)). (4) The pH values expected at *P*_1_ (around 5.63) when adding 10 mM NH_4_Cl (Fig. 1(II)) are somewhat in agreement with the values calculated, being around 5.42 ± 0.01 at −0.35 V, 5.63 ± 0.02 at −0.55 V, and 5.58 ± 0.11 at −0.75 V. These values are only slightly divergent from the values expected. (5) The pH values fitted for the different experiments shown in Fig. 1(III) were also in close agreement. The pH anticipated at *P*_1_ was 5.39, and the values obtained from the experimental curves were 5.44 ± 0.01 at −0.35 V, 5.41 ± 0.02 at −0.55 V, and 5.25 ± 0.11 at −0.75 V.

#### Accumulation of H_2_O_2_

The amount of H_2_O_2_ from the oxygen reduction reaction was measured after one hour of the experiment for each case (Fig. S1 and Table S1, ESI†). Throughout the experiments carried out in this work, hydrogen evolution could be minimized by using carbon gas-diffusion electrodes and tuning the applied potentials that would lead to this reaction. In the NaCl-containing solutions, the concentration of H_2_O_2_ varied from 0.13 mM to 0.69 mM after one hour of the experiment. The maximum concentration of H_2_O_2_ was around 0.69 mM using 0.14 M NaCl + 30 mM NH_4_Cl at −0.75 V (Fig. 1, assay III-c).

#### Current efficiencies (CE%)

The current efficiencies calculated for the generation of H_2_O_2_ are presented in Fig. 1A. Overall, these values are low (between 6% and 11%). When there is no WA present, the most likely side reaction is the four-electron pathway for the ORR (reaction (9)). When the WA is present, the current density is distributed between the four-electron ORR pathway, the generation of H_2_O_2,_ and the deprotonation of the WA.

9 H_2_O_2_ + 2H^+^ + 2e^−^ → 2H_2_O

Overall, there was a slight increase in the H_2_O_2_ concentration, with an increasing concentration of the WA at a constant applied potential that also corresponded to a small increase in the current density. For instance, assays with 10 mM NH_4_Cl (II-b and III-b) achieved a CE% of 7.1% and 7.9% and a current density of around 38 and 53 A m^−2^, respectively. The CE% for assays with 30 mM NH_4_Cl (I-c, II-c, and III-c) was around 9%, 10%, and 11%, whereas the current density was approximately 68, 56, and 63 A m^−2^, respectively. These results show the co-catalytic effect of NH_4_Cl *via* the cathodic deprotonation process, resulting in a higher production rate and concentration of hydrogen peroxide (Fig. 1A).

### Current–potential curves

Current–potential curves were recorded by LSV at the beginning (Fig. 2) and the end (data not shown here) of every experiment to investigate the effect of NH_4_Cl addition. The inset in Fig. 2 shows the full current–potential patterns. The shape of these curves had the same pattern when the experiments were repeated. During the LSV experiments (Fig. 2), the polarization potential was varied from 0 V to −0.75 V using the following background electrolytes: 0.14 M NaCl (black solid line), 0.14 M NaCl + 10 mM NH_4_Cl (red solid line), and 0.14 M NaCl + 30 mM NH_4_Cl (green solid line). In this context, *J*_1_, *J*_2_, and *J*_3_ denote the cathodic current density measured for 0 mM, 10 mM, and 30 mM NH_4_Cl in NaCl solutions, respectively. These LSV results recorded at the start of the experiments suggest that the addition of NH_4_Cl resulted in a consistent small increase in cathodic current density. This can be associated with an increase in the rate of the electrochemical reactions taking place, which in this case led to a higher production of H_2_O_2_ in some assays (II-b, III-b, I-c, II-c, and III-c), which is in good agreement with the slightly increasing concentration of H_2_O_2_ reported in Fig. 1A.

When the pH reached a given alkaline pH value, the increase of current was not obvious as in the LSV recorded before applying the desired potential to start the electrolyses. Therefore, these changes in the behavior of the LSV curves suggest the occurrence of cathodic deprotonation. Thus, it could be inferred from the LSV curves (Fig. 2) recorded at the start of the experiments that NH_4_^+^ ions should be responsible for the current increase as the potential in the LSV curves became more negative during these assays. This effect is not present in LSV curves recorded at the end of experiments (not shown here) wherein the pH is high, and thus protonated species are presumably depleted. The increase in H_2_O_2_ formation thus could be the consequence of the cathodic deprotonation process, as hypothesized. In view of this theory, the protonated species of a buffer (*e.g.*, NH_4_Cl) will lose protons, causing a dependence of the produced current on the concentration of the WA. It should be noted that the electroactive species, electrochemically reduced, are not protons themselves but protonated species, which in turn depend on the dissociation constant of the species and the pH of the solution.^40,42^ Under these experimental conditions, the cathodic deprotonation of NH_4_^+^ ions occurs mostly below and up to a pH close to the p*K*_a_ of NH_4_Cl.

On the other hand, from the modelling of the pH curves presented in Fig. 1 (shown in Fig. S4, ESI†), the amount of NH_4_Cl that seemed to be involved in reaction (8) was lower than the added concentration of either 10 mM or 30 mM in assays II-b, II-c, and III-c. This parameter was only determined in those assays where oxygen was reduced for a sufficient time to effectively induce the reaction between NH_4_^+^ added and the OH^−^ ions formed, to produce the second equivalence point (*P*_2_).

### Formation of IONPs

**Fig. 3:**
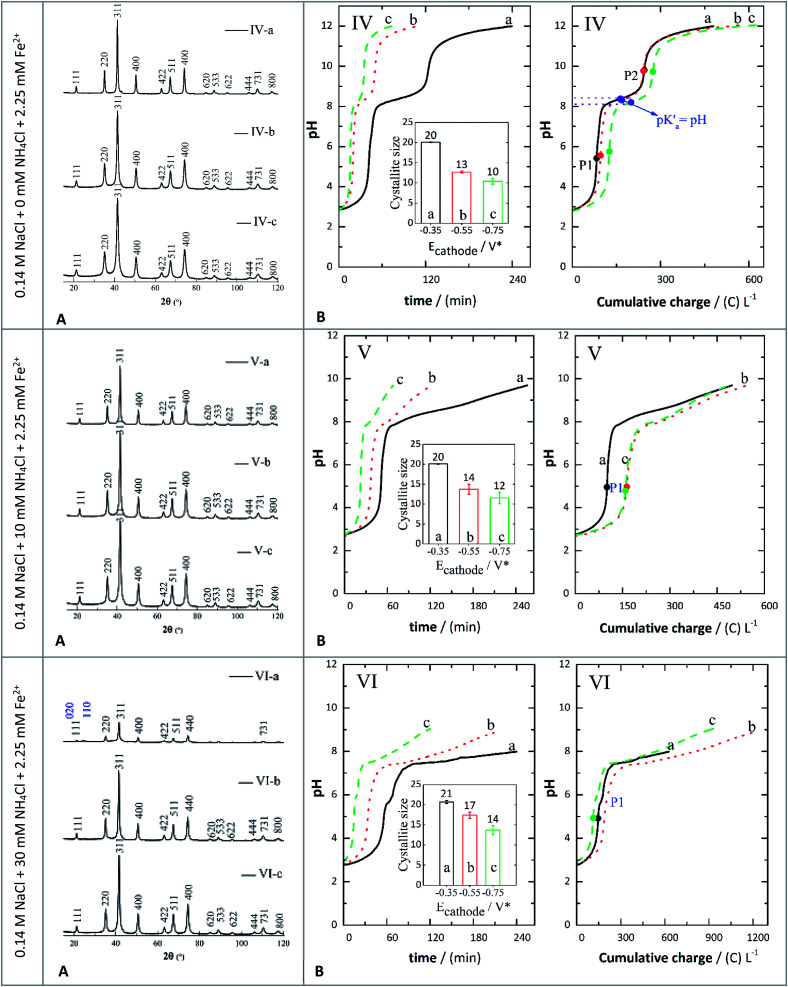
XRD patterns (A) and evolution of pH (B) against either time or charge consumed in 0.14 M NaCl + 2.25 mM Fe^2+^ (IV-a, IV-b, and IV-c), 0.14 M NaCl + 2.25 mM Fe^2+^ + 10 mM NH_4_Cl (V-a, V-b, and V-c), and 0.14 M NaCl + 30 mM NH_4_Cl + 2.25 mM Fe^2+^ (VI-a, VI-b, and VI-c) at different applied potentials of −0.350 V (a), −0.550 V (b), and −0.750 V (c). The insets show the crystallite sizes determined by XRD.

In general, irrespective of the approach used to form the iron oxides of interest, a supersaturation condition is a prerequisite for the precipitation of solids. In turn, such a condition can tailor their size, phase, and other properties.^28^ Thus, a series of electrolysis experiments was performed to prove that the products from the two-electron pathway of the ORR could be used to establish the supersaturation of the solution, its alkalinity, and the necessary redox conditions to obtain stable crystals of iron oxides like magnetite. Besides, the composition of the electrolyte can also influence the final products when they contain inorganic species like chloride ions.^28^ FeCl_2_ was used as the source of Fe^2+^ ions in 0.14 M NaCl to investigate the behaviour of this process, following our previous work.^33^ In addition, in separate experiments, the effect of two different concentrations of NH_4_Cl *i.e.*, 10 mM and 30 mM, respectively, was investigated. These assays are listed in Table 2 and depicted in Fig. 3. The rationale behind this is that the presence of the WA can accelerate the production of H_2_O_2_*via* the cathodic deprotonation mechanism. As shown in our previous research, this should also result in the formation of smaller particles.^44^

**Table 2:** Reaction conditions during the pH evolution of electrolytes with 2.25 mM Fe^2+^

Assay	Electrolyte	Applied potential (V)
IV-a	140 mM NaCl + 0 mM NH_4_Cl + 2.25 mM Fe(ii)	−0.350
IV-b	140 mM NaCl + 0 mM NH_4_Cl + 2.25 mM Fe(ii)	−0.550
IV-c	140 mM NaCl + 0 mM NH_4_Cl + 2.25 mM Fe(ii)	−0.750
V-a	140 mM NaCl + 10 mM NH_4_Cl + 2.25 mM Fe(ii)	−0.350
V-b	140 mM NaCl + 10 mM NH_4_Cl + 2.25 mM Fe(ii)	−0.550
V-c	140 mM NaCl + 10 mM NH_4_Cl + 2.25 mM Fe(ii)	−0.750
VI-a	140 mM NaCl + 30 mM NH_4_Cl + 2.25 mM Fe(ii)	−0.350
VI-b	140 mM NaCl + 30 mM NH_4_Cl + 2.25 mM Fe(ii)	−0.550
VI-c	140 mM NaCl + 30 mM NH_4_Cl + 2.25 mM Fe(ii)	−0.750

### XRD analysis of precipitates

The crystal structures of the prepared samples were characterized by XRD and the results are shown in Fig. 3(A). The peaks indexed (Fig. 3(A)) correspond to crystallographic planes in the following order: (111), (220), (311), (400), (422), (511), (440), (620), (533), (622), (444), (731) and (800). This identification was performed by comparing the XRD diffractograms with reference patterns from the International Centre for Diffraction Data (ICDD) database, and these peak indexes were matched with cards 01-088-0315 (magnetite) and 00-029-0713 (goethite). The XRD patterns showed sharp and intense peaks, suggesting the presence of well-defined crystalline phases, identified mostly as magnetite, except for assay VI-a (Fig. 3) which contained a magnetite-goethite mixture. The latter was identified by the low-angle peaks (020) and (110) marked in Fig. 3(A), corresponding to assay VI-a. Consequently, when the GDEx process was performed by adding 30 mM NH_4_Cl at −0.350 V, crystals of goethite were detected. This is also consistent with the findings of Prato *et al.*^33^

**Fig. 4:**
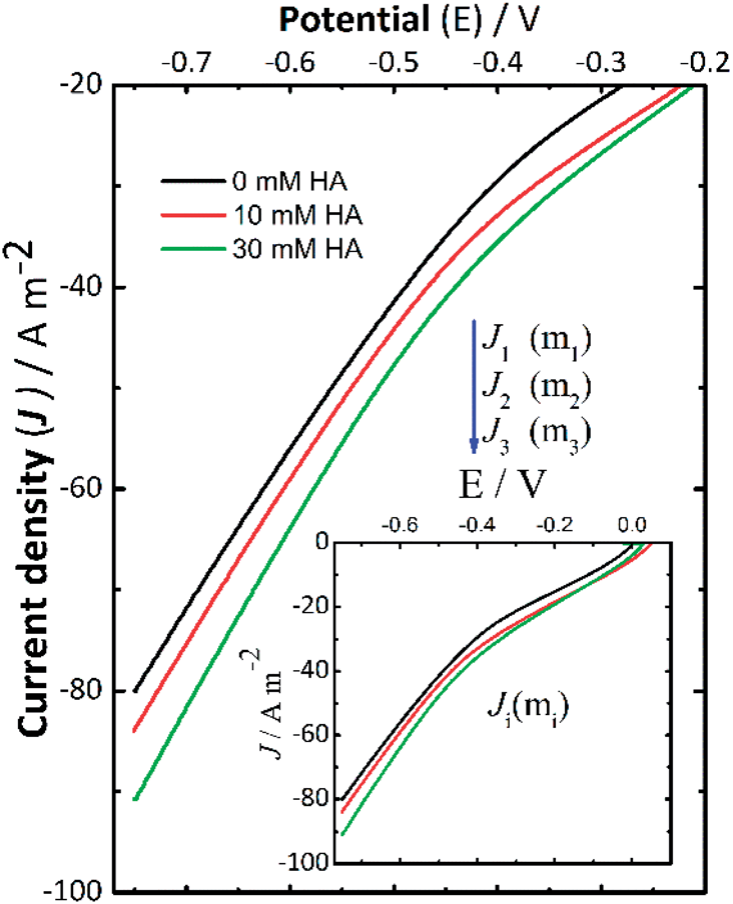
Current–potential curves recorded before starting the electrolysis by linear scan voltammetry (LSV) at a scan rate of 10 mV s^−1^ varying the polarization potential from open circuit potential to −0.75 V *vs.* Ag/AgCl (satd. KCl) in different background electrolytes: 0.14 M NaCl + 2.25 mM Fe^2+^ (black solid line), 0.14 M NaCl + 10 mM NH_4_Cl + 2.25 mM Fe^2+^ (red solid line), and 0.14 M NaCl + 30 mM NH_4_Cl + 2.25 mM Fe^2+^ (green solid line). The inset shows the full current–potential curves.

The average crystal size distribution (insets in Fig. 4B) obtained for the IONPs from XRD was between 12 nm and 20 nm. Specifically, without NH_4_Cl the average sizes were around 20 nm (assay IV-a), 13 nm (assay IV-b) and 10 nm (assay IV-c). When adding NH_4_Cl the average crystal sizes were 20 nm (assay V-a), 14 nm (assay V-b), 12 nm (assay V-c), 21 nm (assay VI-a), 17 nm (assay VI-b) and 14 nm (assay VI-c). It is important to recall here that the indexes *a*, *b* and *c*, correspond to the applied potentials of −0.350 V, −0.550 V, and −0.750 V, respectively. It seems that there is a higher correlation of the crystal size with the applied polarization potential than with the presence or absence of NH_4_Cl. Yet, the influence of adding the WA can still be appreciated. The bigger crystals were on average 20 nm at −0.350 V, irrespective of the addition of WA, but at −0.550 V and −0.750 V slightly smaller crystals were obtained.

This effect was in disagreement with previous research reported where the mechanism through which the crystal size is influenced is as follows: higher supersaturation leads to higher nucleation rates, which in turn generate smaller crystals.^45,46^ This is because when adding the WA the gradual changes in pH presumably favoured the generation of slightly larger crystals.

Magnetite (Fe_3_O_4_) was the only pure phase present at the end of almost all the experiments, except for assay VI-a, as explained before (Fig. 4A). The formation of crystallite domains of Fe_3_O_4_ in this work can be possibly explained by the concomitant formation of oxidising and reducing species like H_2_O_2_. The likely partial oxidation of H_2_O_2_ (ref. 47) *via* either homogeneous Fenton or heterogeneous Fenton-like processes could play an important role in the early stages of formation of the predominant phase of the iron oxide identified at the end of the assays. However, GDEx differs from Fenton or electro-Fenton processes as the latter takes place under acidic conditions (<4), wherein the precipitated products observed in this work would not remain stable.

### pH evolution when adding a metal precursor

In Fig. 3, the pH evolution curves against either time or charge consumed are depicted for the following electrolytes: (IV) 0.14 M NaCl + 10 mM NH_4_Cl + 2.25 mM Fe^2+^, (V) 0.14 M NaCl + 10 mM NH_4_Cl + 2.25 mM Fe^2+^, and (VI) 0.14 M NaCl + 30 mM NH_4_Cl + 2.25 mM Fe^2+^ at the same polarization potentials of −0.350 V (a), −0.550 V (b), and −0.750 V (c) as previously described. At the beginning of each constant polarization experiment, the solutions were colorless and progressively became yellowish. When small precipitates were noticeable, the color transitioned to orange-yellow, ultimately turning into brown-green. The greenish colour may indicate the presence of Fe(OH)_2_.^48^ It then shifted to brown-orange and finally to black, indicating the formation of magnetite (Fe_3_O_4_) (Fig S1, ESI†). However, in some cases the final solution was brown-black, suggesting the formation of more than one phase (Table S2, ESI†).

Because OH^−^ ions were generated continuously, the pH of the electrolyte increased and led to the precipitation of iron hydroxides *via* reactions (10) and (11). In particular, both reactions (10) and (11) are driven by the pH increase near the cathode due to reaction (6).^49,50^ In this regard, the Fe^3+^ ions formed could react with OH^−^ ions to form orange-yellow hydroxides in the solution *via* reaction (10),^23^ whereas Fe^2+^ hydroxides (*i.e.*, Fe(OH)_2_ may completely precipitate at a pH higher than 8.5.^51^ In these experiments, when the electrolyte reached a final pH of 8, in the obtained particles, a mixture of goethite and magnetite was evidenced from the XRD patterns (assay VI-a, Fig. 3(A)). At a final pH around 9 (assays VI-b and VI-c) the prepared crystals were identified as magnetite. Similarly, at a final pH of 9.7 (assays V-a, V-b, and V-c) and pH around 12 (assays IV-a, IV-b, and IV-c), in the dried samples, a single phase corresponding to magnetite was observed.

10 Fe^3+^ + 3OH^−^ → Fe(OH)_3(s)_

11 Fe^2+^ + 2OH^−^ ⇄ Fe(OH)_2(s)_

As reaction (2) took place continuously, the electrogeneration of H_2_O_2_ and OH^−^ also created the necessary supersaturation conditions to transform goethite and Fe(OH)_2_ into magnetite. Reaction (11) was determined to be one of the equilibrium processes depicted in Fig. 3(IV), established between *P*_1_ and *P*_2_. This is because the solubility product of Fe(OH)_2_ is higher than that of Fe(OH)_3_; thus the partial dehydroxylation of Fe(OH)_2_ is favoured as the concentration of OH^−^ ions increased. Furthermore, the pH condition marked as *P*_2_ represents the condition of pH established to form magnetite as a pure phase under the conditions of these assays, which seems to be around a pH of 9.6. In addition, the equivalence points *P*_1_ and *P*_2_ for assays IV-a, IV-b, and IV-c (Fig. 3) were determined using the first and second derivative of the experimental pH data *versus* either time or charge. From the half-point (corresponding to the p*K*_a_) between *P*_1_ and *P*_2_, it was inferred that the equilibrium process of reaction (11) had an average p*K*_a_ of 8.28 ± 0.1 in the systems without the supplemented WA (Fig. 3B-IV).

On the other hand, adjusted curves (shown in Fig. S4, ESI†) to the results in Fig. 3 indicate that the formation of magnetite likely followed the chemical steps in a similar way to what was previously discussed by Lozano *et al.*^29^ Reaction (11) is expressed as reversible to point out that this is the reaction involved in the acid-base equilibrium depicted in Fig. 4B(IV–VI). This equilibrium results from reactions between products of the reduction of oxygen at the cathode as presented at the beginning of the manuscript and the iron species formed, whereas reactions (12) and (13) are likely the chemical steps occurring in the bulk of the electrolyte whose titration curves are depicted in Fig. 3B.

Lozano *et al.*^29^ stated that Fe(OH)_2_, under alkaline conditions and in the presence of air, can be transformed into γ-FeOOH (lepidocrocite phase) that finally reacted with additional Fe(OH)_2_ to form magnetite *via* a topotactic process.^29^ This pathway seemed to be promoted by the presence of a high concentration of Fe^2+^ ions, as their study involved the anodic dissolution of a sacrificial iron anode. Nonetheless, in this research, the concentration of Fe^2+^ ions was limited by an initial amount added to the electrolytes as shown in Fig. 3. This lack of excess Fe^2+^ ions likely promoted an alternative mechanism to form magnetite, which did not involve lepidocrocite as the main intermediate as proposed by Lozano *et al.*^29^ Instead, goethite (α-FeOOH_(s)_) was identified by XRD as a precursor in our systems as discussed before. This phase has been obtained by direct transformation of Fe(OH)_2_ under highly alkaline conditions at pH values between 12.8 and 12.3.^52^ It has also been previously obtained through GDEx under a specific set of experimental conditions, mainly related to the concentration of Fe^2+^ in solution and the charge applied in GDEx.^33^ In this research the main transformations were observed at pH values lower than those reported by Gilbert *et al*.^52^ Hence, the formation of goethite is inferred to be the result of the slow hydrolysis of Fe^3+^ hydroxy species.^53^ These species could act as seeds to direct the transformation of FeOOH_(s)_ formed *via* partial oxidation of Fe(OH)_2_ by H_2_O_2_, as shown in reaction (12). Finally, α-FeOOH_(s)_ and Fe(OH)_2_ could react in a topotactic process to form magnetite *via* reaction (13) as previously discussed for other FeOOH polymorphs.^29,52,54,55^

12 Fe(OH)_2(s)_ + H_2_O_2_ → FeOOH_(s)_ + H_2_O

13 2α-FeOOH_(s)_ + Fe(OH)_2(s)_ → Fe_3_O_4(s)_ + 2H_2_O

### Current–potential curves

Current–potential curves were recorded before starting and at the end of each electrolysis by LSV (Fig. 4). The aim was to investigate the effect of adding Fe^2+^ ions compared to the cathodic deprotonation effects previously presented for the ORR to H_2_O_2_ described in Fig. 3, from 0 V to −0.75 V. The insets correspond to the full current–potential curves using the following background electrolytes: 0.14 M NaCl + 2.25 mM Fe^2+^, 0.14 M NaCl + 10 mM NH_4_Cl + 2.25 mM Fe^2+^, and 0.14 M NaCl + 30 mM NH_4_Cl + 2.25 mM Fe^2+^. These LSV curves show the same changes when adding NH_4_Cl, either with or without Fe^2+^. The open-circuit potential for these curves was around −0.24 ± 0.01 V. As mentioned before, *J*_1_, *J*_2,_ and *J*_3_ denote the cathodic current density measured for the assays described in Table 2, using NaCl as the background electrolyte (Fig. 4). The LSV curves recorded showed a similar behavior to those in Fig. 2, where the addition of NH_4_Cl (from 10 mM to 30 mM) resulted in a consistent small increase in cathodic current density. As discussed earlier, this is a relevant outcome and has favourable implications for the formation of the crystallite domains of iron because the gradual changes in pH can allow conditions for the slow oxidation of intermediates to form pure phases like magnetite. However, it is important to bear in mind that appropriate selection of the added WA should be accompanied by the analysis of the chemical nature of the species of interest to ultimately produce suitable supersaturation conditions (*i.e.*, temperature, applied potential or current density).

Recovery efficiencies of iron (*η*).The recovery efficiencies from the assays described in Fig. 3 were determined by monitoring the concentration of total iron dissolved in the solution at the beginning and at the end of each assay by ICP-MS. From these analyses, it was established that an average recovery of 99.9 ± 0.2% of the total iron added was obtained in the aqueous electrolyte. Thus, GDEx provides an effective route to recovering soluble iron species to IONPs.

## Conclusions

The buffering ability of NH_4_Cl in aqueous solutions of 0.1 M NaCl enhanced the generation of H_2_O_2_*via* the cathodic deprotonation process. The applied polarization potential had a greater influence on the size of the crystals of iron measured. This effect was exceptionally noticeable in almost all instances where magnetite (Fe_3_O_4_) was identified at a final pH higher than 8.5. Otherwise, a mixture of magnetite-goethite was evidenced at lower pH values. The chemical reactions occurring to continuously form H_2_O_2_ and OH^−^ facilitated the necessary conditions of supersaturation, leading to the production of smaller crystals. This study demonstrates the feasibility of the method in preparing nano-sized magnetite particles, with *in situ* generated oxidizing species in aqueous solutions. Moreover, it establishes GDEx as an effective strategy to completely deplete iron ions in the solution, with prospective applications in metal recovery and remediation.

## Conflicts of interest

There are no conflicts to declare.

## Supplementary Material

NA-002-C9NA00738E-s001
