# Supplementary material for: pH Transitions and electrochemical behavior during the synthesis of iron oxide nanoparticles with gas-diffusion electrodes
Source: Nanoscale Adv. 2020 Feb 13;2(5):2052–62. doi: 10.1039/c9na00738e (PMC9419531; doi:10.1039/c9na00738e)
Supplement: NA-002-C9NA00738E-s001 [file NA-002-C9NA00738E-s001.pdf]

Electronic Supplementary Material (ESI) for Nanoscale Advances.
This journal is © The Royal Society of Chemistry 2020

Electronic Supplementary Information for Nanoscale Advances manuscript:

pH Transitions and electrochemical behavior during the synthesis of iron oxide nanoparticles with gas-diffusion electrodes

Burgos-Castillo Rutely C.,[ab‡] Garcia-Mendoza Arturo,[c] Alvarez-Gallego Yolanda,[ad] Fransaer Jan,[de] Sillanpää Mika[b] and Dominguez-Benetton Xochitl[ad]*

[a.] *Separation and Conversion Technologies, Flemish Institute for Technological Research (VITO), Boeretang 200, 2400, Mol, Belgium.*
[b.] *Department of Green Chemistry, School of Engineering Science, Lappeenranta University of Technology - Sammonkatu 12, FI-50130 Mikkeli, Finland.*
[c.] *Departamento de Química Analítica, Facultad de Química, Universidad Nacional Autónoma de México, Av. Universidad 3000, C.U. Mexico City, 04510, Mexico.*
[d.] *SIM vzw, Technologiepark 935,BE-9052 Zwijnaarde, Belgium.*
[e.] *Department of Materials Engineering, Katholieke Universiteit Leuven (KU Leuven), Kasteelpark Arenberg 44 - bus 2450, B-3001 Leuven, Belgium.*

* xoch@vito.be
‡ rcbcastillo@gmail.com
† *Electronic Supplementary Information (ESI) available.*

Table S1. The reaction conditions during the pH evolution

System	Electrolyte	Applied potential / mV	$[H_2O_2]$ / µM	Charge / C	pH	Final pH
I-a	140 mM NaCl	−350	132	115	11.3	11.7
II-a	140 mM NaCl + 10 mM NH_4Cl	−350	252	115	8.9	9.5
I-b	140 mM NaCl	−550	441	300	11.8	12.0
II-b	140 mM NaCl + 10 mM NH_4Cl	−550	441	300	9.8	11.5
III-b	140 mM NaCl + 30 mM NH_4Cl	−550	518	314	8.8	9.3
I-c	140 mM NaCl	−750	581	300	11.8	12.0
II-c	140 mM NaCl + 10 mM NH_4Cl	−750	635	300	9.8	11.7
III-c	140 mM NaCl + 30 mM NH_4Cl	−750	687	301	9.1	10.0

Table S2. Crystallite size and phases identified in this study from XRD spectra

Assay	Potential / V	Final pH	Crystallite size / (nm) by XRD	Phase(s)	Electrolyte
Fig. 4-IV-a	-0.350	11.7 (120 min)	20.1 ± 0.1	Magnetite	140 mM NaCl + 0 mM NH_4Cl + 2.25 mM Fe(II)
Fig. 4-IV-b	-0.550	12.0 (90 min)	13.8 ± 1.3	Magnetite	140 mM NaCl + 0 mM NH_4Cl + 2.25 mM Fe(II)
Fig. 4-IV-c	-0.750	11.9 (60 min)	11.6 ± 1.4	Magnetite	140 mM NaCl + 0 mM NH_4Cl + 2.25 mM Fe(II)
Fig. 4-V-a	-0.350	9.7 (256 min)	19.6 ± 0.2	Magnetite	140 mM NaCl + 10 mM NH_4Cl + 2.25 mM Fe(II)
Fig. 4-V-b	-0.550	9.7 (121 min)	14.6 ± 0.9	Magnetite	140 mM NaCl + 10 mM NH_4Cl + 2.25 mM Fe(II)
Fig. 4-V-c	-0.750	9.7 (68 min)	12.9 ± 0.5	Magnetite	140 mM NaCl + 10 mM NH_4Cl + 2.25 mM Fe(II)
Fig. 4-VI-a	-0.350	8.0 (240 min)	20.7 ± 0.4	Magnetite Goethite	140 mM NaCl + 30 mM NH_4Cl + 2.25 mM Fe(II)
Fig. 4-VI-b	-0.550	8.9 (210 min)	17.4 ± 0.8	Magnetite	140 mM NaCl + 30 mM NH_4Cl + 2.25 mM Fe(II)
Fig. 4-VI-c	-0.750	9.04 (120 min)	13.7 ± 1.1	Magnetite	140 mM NaCl + 30 mM NH_4Cl + 2.25 mM Fe(II)

Table S3. Current efficiency calculations

System	Description	[1]Q_{analyt} (C)	Q_{exp} (C)	CE(%)
I-a	140 mM NaCl	6.1	114.9	5.3
I-b	140 mM NaCl	21.3	300.3	7.1
I-c	140 mM NaCl	28.0	300.3	9.3
II-a	140 mM NaCl + 10 mM NH_4Cl	6.4	114.9	5.5
II-b	140 mM NaCl + 10 mM NH_4Cl	21.3	300.3	7.1
II-c	140 mM NaCl + 10 mM NH_4Cl	30.6	300.1	10.2
III-a	140 mM NaCl + 30 mM NH_4Cl	3	314.2	3.2
III-b	140 mM NaCl + 30 mM NH_4Cl	25.0	314.2	7.9
III-c	140 mM NaCl + 30 mM NH_4Cl	33.1	300.6	11.0

The current eficiency (*CE*(%)) is a yield based on the total electrochemical charge that is consumed during the electrolysis. In other words is the efficiency to selectively transfer electrons flowing through the electrochemical circuit to the product of interest (measured analytically). Q_{analyt} is the amount of charge that is consumed in forming H_2O_2, whereas Q_{exp} is the total charge transferred during the electrolysis. Here, *CE* is defined as the ratio between Q_{analyt} and Q_{exp}. Note that Q_{analyt} equals to the well-known term $n{\cdot}F{\cdot}V{\cdot}C$ (derived from Faraday's Law). Where n is the number of electrons transferred per mol of electrogenerated species (2 e^- per mol), F is the Faraday's constant (96,485 C mol^{-1}), V is the volume of the solution (L), C is the concentration of H_2O_2 determined experimentally (mol L^{-1}). Q_{exp} is the total charge consumed in one hour during the experiments.

Table S4. Summary of parameters obtained by the non-linear adjustments presented in Figure S4 in the ESI of this the manuscript. The last three columns present data taken from Figure 2 of the manuscript and are added for the purpose of comparison.

Assay	$[NH_4Cl]$ / mM	[HCl] / mM	J/ A m^{-2}	R^2	pK_a NH_4^+/NH_3	pH at P1	pH at P2	E / V	$[H_2O_2]$/ mM	CE/(%)
**I-a**	0.0	0.97	19.48	0.99675		≈7.00	-	-0.35	0.13	5.5
**II-a**	10.0	1.19	23.34	0.99978	9.38	5.69	10.69	-0.35	0.13	5.5
**III-a**	30.0	2.18	22.88	0.99581	9.38	5.45	10.93	-0.35	0.06	3.3
**I-b**	0.0	0.62	48.19	0.99466		≈7.00	-	-0.55	0.44	7.1
**II-b**	7.1	0.79	37.92	0.99989	9.45	5.80	10.65	-0.55	0.44	7.1
**III-b**	30.0	1.54	53.21	0.99374	9.38	5.45	10.93	-0.55	0.52	7.9
**I-c**	0.0	0.85	68.20	0.99399		≈7.00	-	-0.75	0.58	9.3
**II-c**	7.0	0.75	55.78	0.99991	9.47	5.81	10.66	-0.75	0.63	10.2
**III-c**	17.4	0.65	62.76	0.99986	9.49	5.62	10.87	-0.75	0.69	11

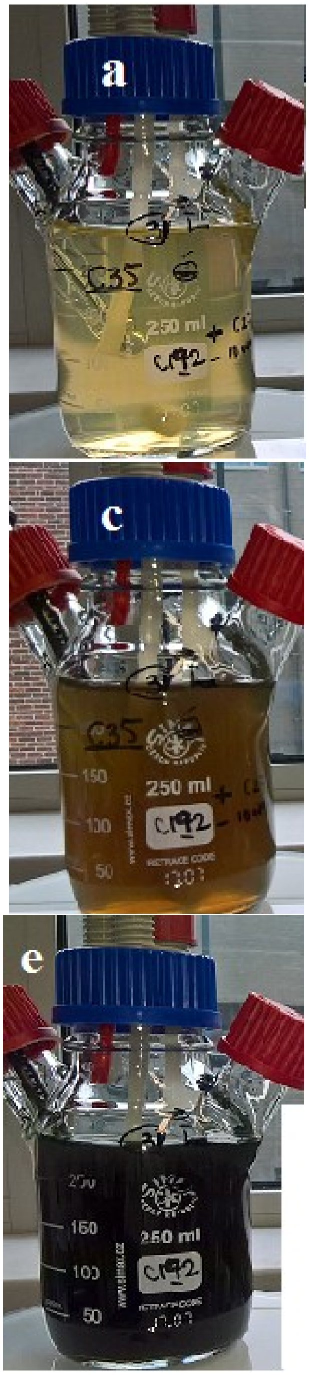

Figure S1. The color of the reaction solution at different times along the pH evolution of the experiments for either a), c), e) 0.14 M NaCl.

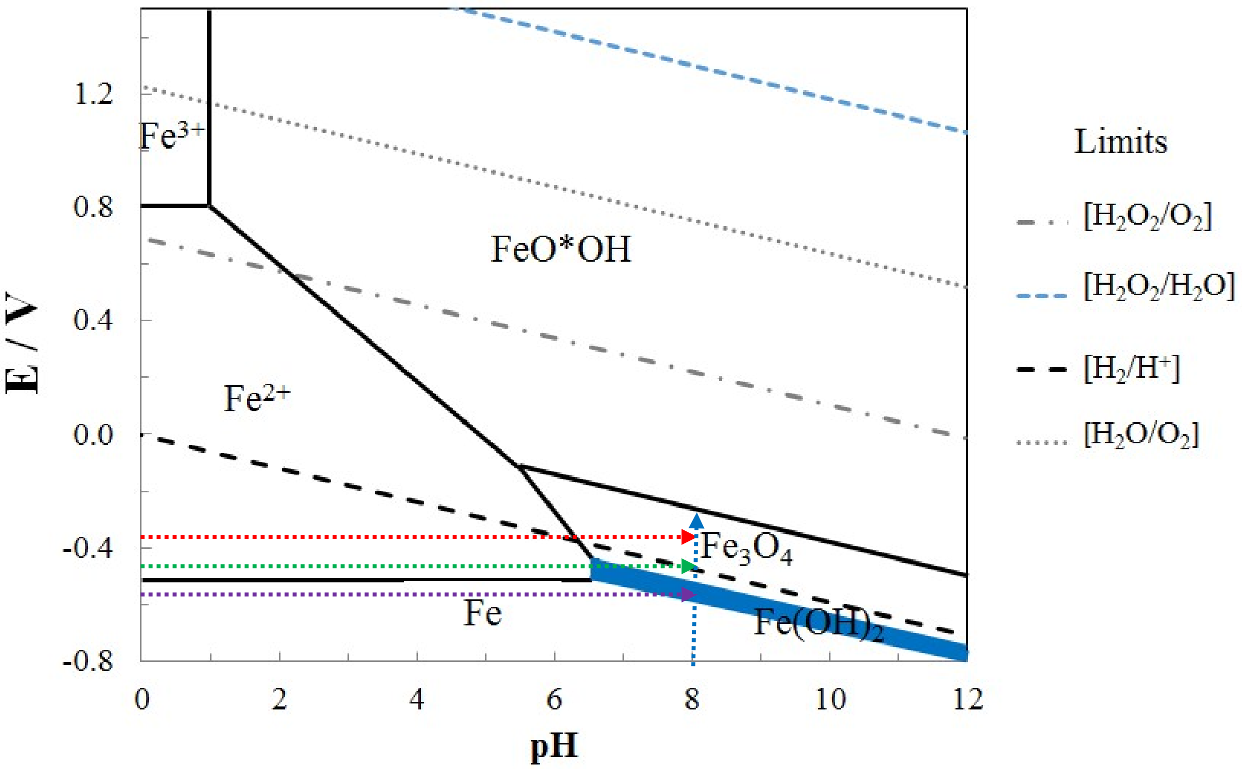

Figure S2. E-pH diagram for aqueous species in the system Fe-H_2O in the presence of H_2O_2, in a NaCl supporting electrolyte, indicating the stable equilibria at 22 ° C and 1 atm. Different molar concentration of the dissolved species were considered, as pertinent to the present study.

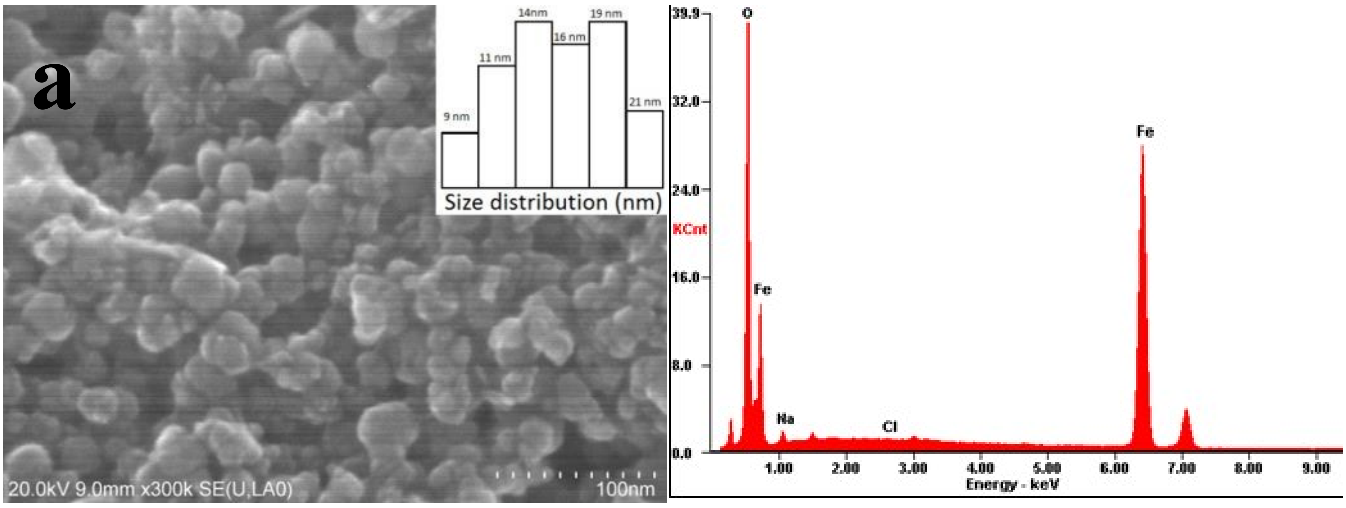

Figure S3. SEM images of selected samples. The images depict spherical-shaped nanoparticles for 0.14 M NaCl + 30 mM NH_4Cl at -750 mV. The nanoparticle morphology is representative of all iron oxide samples obtained.

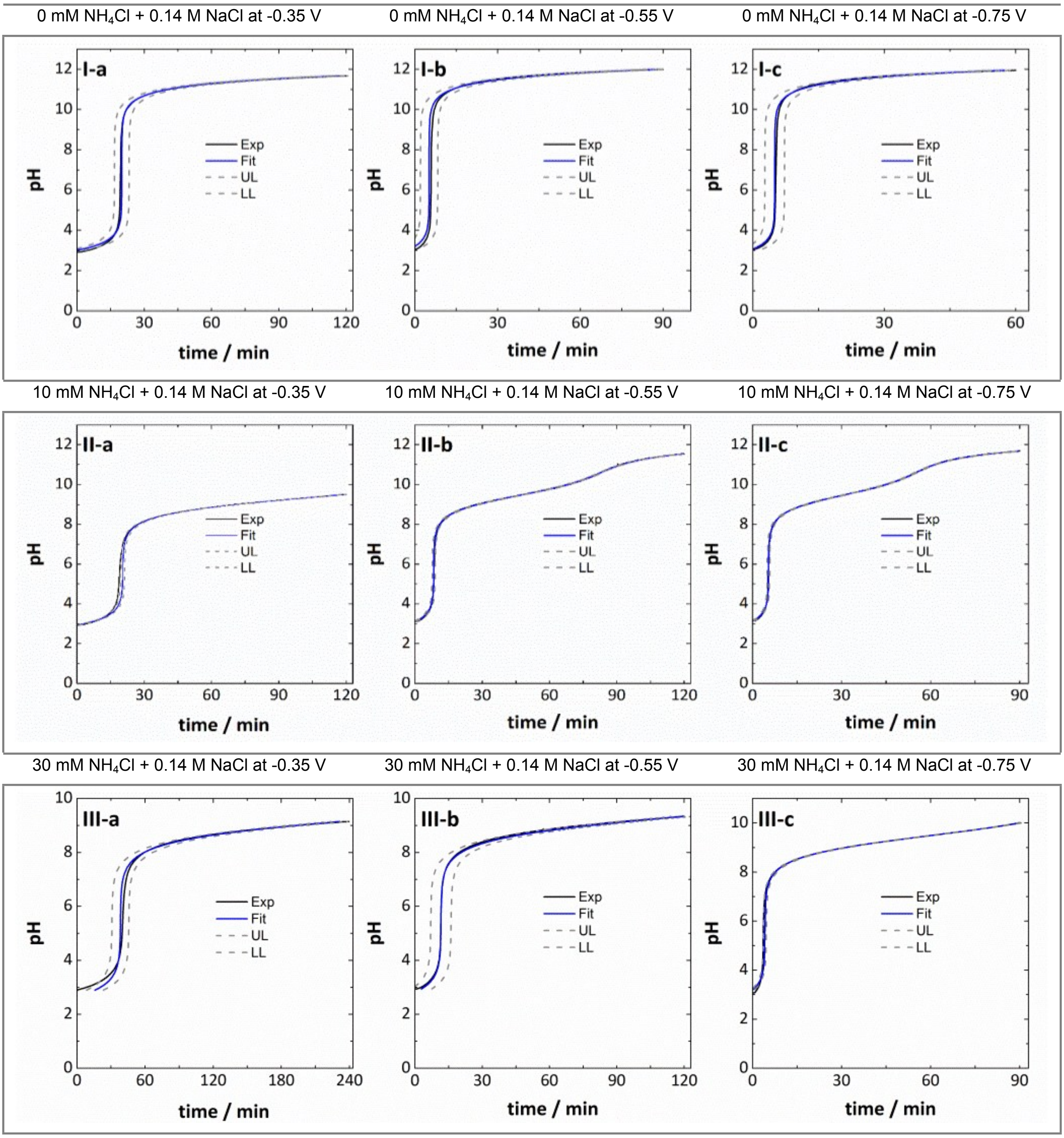

Figure S4. pH against time curves fitted for different background electrolytes using a confidence interval of 95%: (I) 0.14 M NaCl. (II) 0.14 M NaCl + 10 mM NH_4Cl. (III) 0.14 M NaCl + 30 mM NH_4Cl at different applied potentials of (a)-0.35 V; (b)-0.55 V; and (c) -0.75.

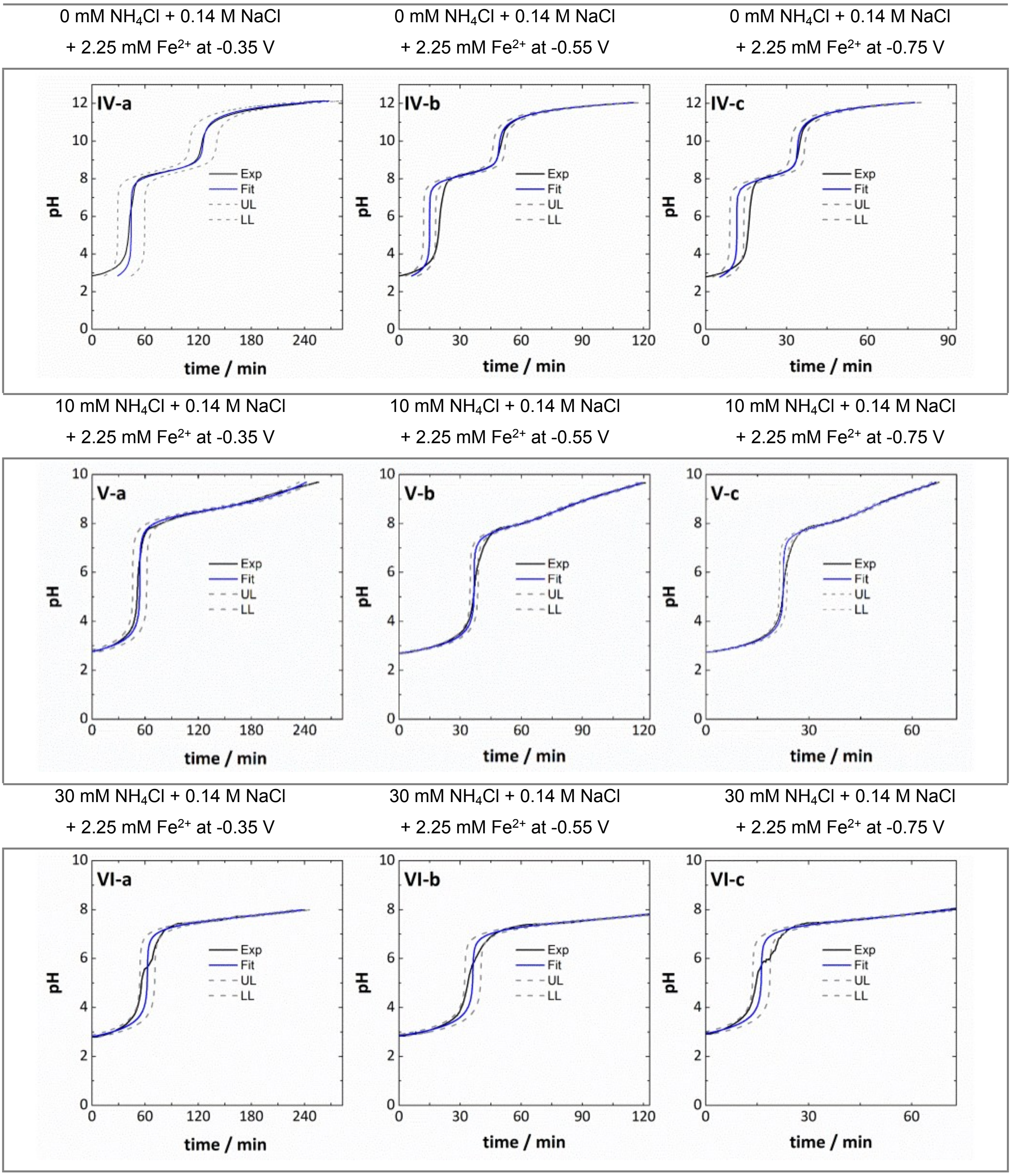

Figure S5. pH against time curves fitted for different background electrolytes using a confidence interval of 95%: (I) 0.14 M NaCl + 2.25 mM Fe^{2+}. (II) 0.14 M NaCl + 10 mM NH_4Cl + 2.25 mM Fe^{2+}. (III) 0.14 M NaCl + 30 mM NH_4Cl + 2.25 mM Fe^{2+}; at different applied potentials of (a)-0.35 V; (b)-0.55 V; and (c) -0.75.
